# Cigarette smoking is associated with high level of ferroptosis in seminal plasma and affects semen quality

**DOI:** 10.1186/s12958-020-00615-x

**Published:** 2020-05-27

**Authors:** Zhanhui Ou, Qirong Wen, Yu Deng, Yang Yu, Zhiheng Chen, Ling Sun

**Affiliations:** 1 Center of Reproductive Medicine, Guangzhou Women and Children’s Medical Center, Guangzhou Medical University, 510623, Guangzhou, Guangdong P.R. China; 2 Department of Gynecological Oncology, Guangzhou Women and Children’s Medical Center, Guangzhou Medical University, 510623, Guangzhou, Guangdong P.R. China

**Keywords:** Ferroptosis, Cigarette smoking, Semen quality, Cigarette smoke condensate, Infertility

## Abstract

**Purpose:**

The effects of cigarette smoking on male semen quality are controversial, and the molecular mechanisms underlying how cigarette smoking affects semen quality are not clear yet.

**Methods:**

In this study, semen samples from 70 heavy smokers and 75 non-smokers receiving infertility treatment were included. Basic semen parameters in non-smokers and heavy smokers were evaluated. Levels of glutathione (GSH), lipid reactive oxygen species (ROS), iron and GSH-dependent peroxidase 4 (GPX4) protein level were observed in human seminal plasma and in GC-2*Spd* cells exposed to cigarette smoke condensate (CSC).

**Results:**

Heavy smokers had significantly higher abnormalities (sperm viability and sperm progressive motility) than non-smoking counterparts. Comparing non-smokers group, GSH level was reduced in the group of heavy smokers (*P* < 0.05). However, the level of lipid ROS and iron were significantly increased (P < 0.05). Besides, GSH level was reduced following treatment with CSC for 24 h, while lipid ROS and iron levels were increased (*P* < 0.05). However, the levels were reduced after being co-cultured with Ferrostatin-1 (Fer-1) (P < 0.05). The level of GPX4 protein was reduced after being treated with CSC in 24 h, and increased after being co-cultured with Fer-1(*P* < 0.05).

**Conclusion:**

Cigarette smoking is associated with high level of ferroptosis in seminal plasma and affect semen quality.

## Introduction

Infertility affects 10–15% of couples worldwide and has become a public health problem in recent years [1]. Male infertility accounts for approximately half of these problems, and decreased semen quality has been widely reported in recent years [2, 3]. The most common cause of male infertility is defective sperm function, which may be affected by genetic disorders, genital tract infections, medical interventions, environmental contamination. Cigarette smoking is also considered to be a risk to male infertility as it could be directly and indirectly toxic to spermatogenesis [4–6]. Even though the reason for the decline of semen quality remains unknown, the correlation between smoking and semen quality has been reported in many studies [7–11]. However, the effect of cigarette smoking on sperm quality reported by some studies was shown to be limited [12, 13]. Therefore, the effect of cigarette smoking on semen quality and its mechanism needs to be clarified.

Cigarette smoke contains high concentrations of nitric oxide, peroxynitrite, and free radicals which can potentially induce the production of cellular reactive oxygen species (ROS) in the human body [14, 15]. Thus, this might be the main reason for the decreased semen quality caused by cigarette smoking, as some studies have also reported [4, 16]. Moreover, it has also been reported that the basic semen parameters could be influenced by cigarette smoking [17–19]. Based on these findings, we believe that cigarette smoking could impair the sperm quality, but what is its potential mechanism?

Ferroptosis is an identified and non-apoptotic regulated cell death with dependency of iron and lipotoxicity, which is firstly observed in cancer cells with oncogenic Ras mutation and could be induced by erastin [20]. Ferroptosis occurs depending on the process of lipid peroxidation activated by non-enzymatic (Feton reactions) and enzymatic mechanism (lipoxygenases) [21]. It is characterized by a production of reactive oxygen species from accumulated iron and lipid peroxidation [22]. Ferroptosis can be induced in the classic way of the inactivation of GPX4, which is the major protector of cellular peroxidation damage [23]. One of the main mechanisms of inactivation of GPX4 is the deprivation of glutathione (GSH) [23]. It can be said that the likelihood of ferroptosis is determined by the balance between ROS production and peroxidation-antioxidant system [24]. This imbalance can result in a number of diseases, including hemochromatosis, neurotoxicity, cancer, liver injury and so on [22, 25–27]. Furthermore, Li et al. showed ferroptosis was a pervasive and dynamic type of cell death induced by oxygen-glucose deprivation and reoxygenation injury in Sertoli cells [22, 25–27], Bromfield also showed oxidative stress induces ferroptosis in spermatids [28]. In summary, we hypothesize that cigarette smoke might reduce the semen quality via the inducing lipid peroxidation and the further occurrence of ferroptosis.

In this study, we first collected the semen samples from heavy smokers and non-smokers and evaluated the semen quality via comparing the parameters. Moreover, the occurrence of ferroptosis was also detected in seminal plasma and GC-2*spd* cell line after the treatment of cigarette smoke condensate, while also detecting the level of GPX4 in the GC-2*spd* cell, which is the key regulator of ferroptosis. We aim to find out the potential mechanism of the sperm impairment caused by cigarette smoking.

## Materials and methods

### Study subjects

This study was declared exempt by the ethics committee of the Guangzhou Women and Children’s Medical Center. All studies involving human subjects were conducted in accordance with guidelines laid down in the Declaration of Helsinki. Written informed consents were obtained by the participants, and the subjects’ data on medical history, lifestyle, and smoking status with a structured questionnaire were collected. Individuals who had never smoked were defined as non-smokers. Current smokers with smoking dose more than 1 pack per day over 10 years or smoking dose more than 2 packs per day over 5 years were defined as heavy smokers. They were men who visited the reproductive medicine center in the Guangzhou Women and Children’s Medical Center for infertility treatment during March 2017 to December 2017. Non-smokers were randomly selected from patients who were age-matched to heavy smokers. All subjects underwent physical examinations and at least two semen analyses. Men who were unhealthy or had a known cause of defective spermatogenesis, such as varicocele, infection, obstruction of the vas deferens, chromosomal abnormalities, or microdeletions of the azoospermia factor region on the Y chromosome were excluded. Patients who were diagnosed with azoospermia, severe oligozoospermia (sperm concentration < 5*10^6^ cells/mL), hemospermia, leukospermia, and necrozoospermia were also excluded. Finally, 70 heavy smokers and 75 non-smokers were recruited.

### Semen collection and analysis

Semen samples were collected in sterile containers from patients by masturbation after 2–7 days of sexual abstinence. Samples were allowed to liquefy for at least 30 min at room temperature. Analysis of semen volume, pH, sperm concentration, motility, vitality, sperm morphology, and computer-assisted semen analysis were carried out according to WHO guidelines [29]. Samples were centrifuged at 1000 *3 g for 10 min, and seminal plasma and cell pellets were separated and stored at − 80 °C until analysis.

### Cell culture and cigarette smoke condensate treatment

GC-2*spd* cell lines were purchased from the American Tissue Culture Collection (ATCC) and maintained in Dulbecco modified Eagles media (DMEM, Gibco, USA) supplemented with heat-inactivated 10% fetal bovine serum (Biological Industries, Israel) in a 95% air-humidified incubator with 5% CO_2_ at 37 °C as our previous study [30].

Cigarette smoke condensate (CSC) was prepared according to the study [31] and resuspended at a concentration of 50 mg/mL in dimethyl sulfoxide (DMSO) as stock solution. For smoke-exposure experiments, cells were cultured in medium with 400 μg/mL of CSC (after treated with 0–800 μg/mL of CSC for 24 h). After exposure to CSC for 24 h, cells were used for further study.

### Cell viability assay

Cell viability was determined with a Cell Counting Kit-8 (CCK-8) assay. Briefly, cells were plated at a density of 5 × 10^4^ cells/well in a 96-well plate with 100 μl of medium. Following treatment, cell viability was evaluated with CCK-8 according to manufacturer’s instructions, and absorbance was measured at 450 nm with a microplate reader. Cell viability was expressed as the percentage of live cells vs. controls (set at 100%).

### Iron assay

The relative iron concentration in cell lysates was assessed using the Iron Assay Kit (no. ab83366; Abcam) according to the manufacturer’s instructions and our previous study [32].

### Lipid peroxidation assay

The relative malondialdehyde (MDA) concentration in cell lysates was assessed using a Lipid Peroxidation (MDA) Assay Kit (no. ab118970; Abcam) according to the manufacturer’s instructions.

### Glutathione assay

The relative glutathione (GSH) concentration in cell lysates was assessed using the Glutathione Assay Kit (no. CS0260; Sigma) according to the manufacturer’s instructions.

### Western blot analysis

Following treatment, cells were washed twice with cold PBS and extracted with RIPA lysis buffer on ice. Extracted cells were then sonicated and centrifuged at 15,000 g for five minutes. Protein extraction was carried out in accordance with existing protocols (Beyotime, China). Protein content was determined using a BCA protein assay kit, according to manufacturer’s instructions. Equivalent amounts of protein were separated on 10–15% SDS-polyacrylamide gels and blotted onto a nitrocellulose membrane. After blocking at room temperature for two hours with 5% non-fat milk in TBS with 0.1% Tween-20, membranes were probed with GPX4 (1:5000 dilution), GAPDH (1:5000 dilution) and HRP conjugated IgG antibodies (1:10,000 dilution); and visualized by exposure to Gel Doc XR (BioRad, USA). Relative band intensity was then determined by standard scanning densitometry normalized with GAPDH.

### Statistical analysis

The independent sample t-test was used to analyze numerical data in two groups. The nonparametric Mann-Whitney test was used to analyze differences between nonhomogeneous variances. One-way ANOVA with Tukey post-test was used to analyze data in multiple groups (Cell viability, GSH, lipid ROS and iron level in the GC-*2spd* cells) (SPSS version 17.0 for Windows). A two-sided *P* value of 0.05 was considered statistically significant.

## Results

### Semen parameters of study subjects

The mean age and semen parameters of 75 non-smokers and 70 heavy smokers are given in Table 1. The two groups had similar average age (34.4 years vs. 35.8 years). The sperm vitality (65.4% vs. 59.4%), and sperm progressive motility (45.5% vs. 36.9%) between the two groups were significantly different (*P* < 0.05), but other basic semen parameters, including sperm concentration (64.6 *10^6^ /mL vs. 56.5 *10^6^ /mL), semen volume (3.2 mL vs. 3.2 mL), abnormal sperm morphology (95.1% vs. 96.1%), and sperm counts (216.5 *10^6^ ells vs. 189.6 *10^6^ cells) were not significantly different (*P* > 0.05) (Table 1).

**Table 1:** The semen parameters of 75 non-smokers and 70 heavy smokers

Parameter	Non-smokers	Heavy smokers	P
N	75	70	
Age (y)	34.4 ± 5.7	35.8 ± 4.5	0.101
Sperm volume (mL)	3.2 ± 1.1	3.2 ± 1.2	0.836
Sperm concentration (X10^6^)	64.6 ± 15.6	56.5 ± 35.4	0.081
Sperm count (X10^6^ cells/mL)	216.5 ± 134.3	189.6 ± 152.5	0.262
Sperm progressive motility (%)	45.5 ± 12.2	36.9 ± 15.7	0.000
Abnormal sperm morphology (%)	95.1 ± 3.2	96.1 ± 1.8	0.230
Sperm vitality (%)	65.4 ± 14.8	59.4 ± 17.9	0.029

Note: Data are presented as mean ± SD

### Seminal plasma GSH, lipid ROS and iron level

**Fig. 1:**
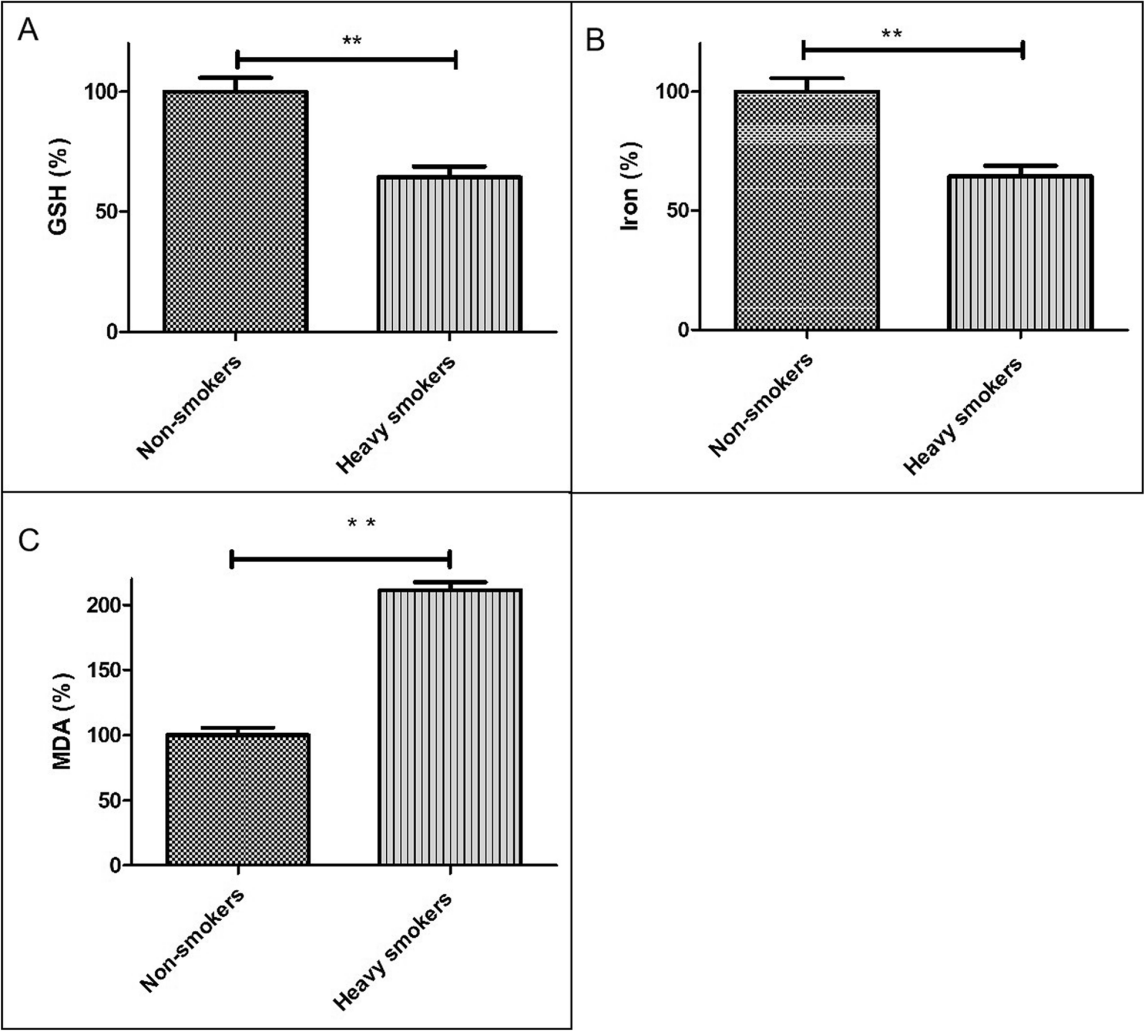
Seminal GSH, lipid ROS and iron level in non-smokers and heavy smokers. **a** GSH level in the semen of non-smokers and heavy smokers. **b** Iron level in the semen of non-smokers and heavy smokers. **c** Lipid ROS level in the semen of non-smokers and heavy smokers. *P < 0.05,**P < 0.01 represents the statistical difference compared to the respective group (*n* = 3)

To investigate the ferroptosis level between non-smokers and heavy smokers, GSH, lipid ROS and iron level were detected in the seminal plasma. Comparing non-smokers group, GSH level was reduced in the group of heavy smokers (*P* < 0.05). However, the level of lipid ROS and iron were increased (P < 0.05) (Fig. 1a, b, c). Collectively, these indicated that ferroptosis level was increased in the seminal plasma of heavy smokers.

### Cell death level in GC-2*spd* cells after CSC treatment

**Fig. 2:**
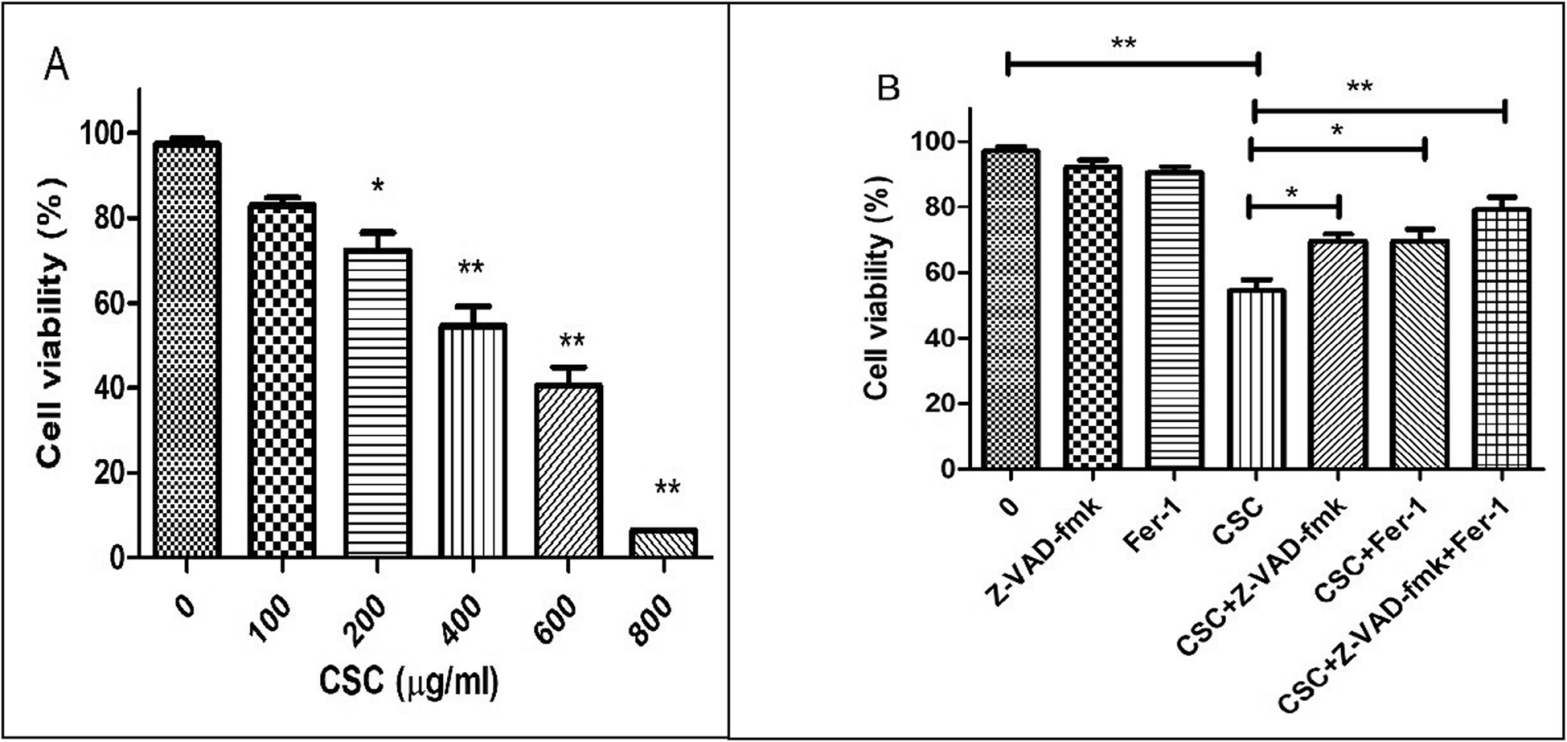
Exposure to CSC-triggered cell death in GC-2*spd* cells. **a** GC-2*spd* cells were treated with 0–800 μg/mL of CSC for 24 h. Cell viability was measured by CCK-8 assay (n = 3). **b** The viability of GC-2*spd* cells were detected after treatment with 400 μg/mL of CSC, we also detected the cellular viability after the treatment with Fer-1 and Z-VAD-fmk to eliminate their cytotoxicity. And the cellular viability following pre-incubation with 400 μg/mL of CSC plus 10 μmol/L of Fer-1, Z-VAD-fmk and Fer-1 combined with Z-VAD-fmk for 24 h was detected respectively. *P < 0.05,**P < 0.01 represents the statistical difference compared to the respective group (n = 3)

The effect of CSC on the viability of GC-2*spd* cells was investigated using the CCK-8 assay. Cell viability was reduced in a dose-dependent manner following treatment with CSC in 24 h (Fig. 2a). To determine whether CSC-induced toxicity was caused by apoptosis or/and ferroptosis independent, Z-VAD-fmk (a broad caspase spectrum inhibitor) and Ferrostatin-1 (Fer-1, an ferroptosis inhibiter) were co-culture with CSC. And the level of CSC-induced toxicity was reduced, but still not totally inhibited by the co-treatment of Z-VAD-fmk (*P* < 0.05). Next, Ferrostatin-1and Z-VAD-fmk were co-culture with CSC. The level of CSC-induced toxicity was reduced dramatically (*P* < 0.01) (Fig. 2b).

### Ferroptosis level in GC-2*spd* cells after CSC treatment

**Fig. 3:**
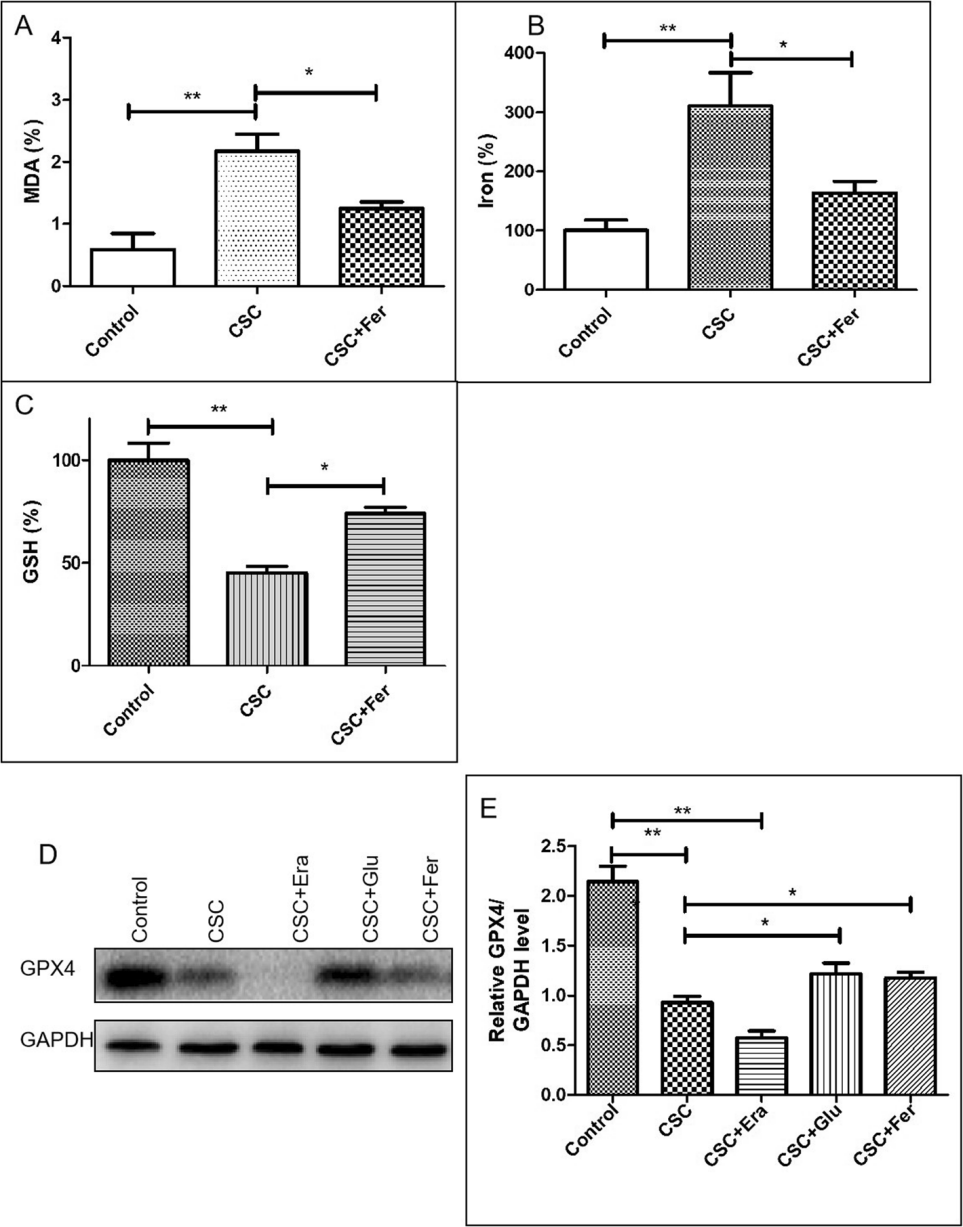
GSH, lipid ROS and iron level in GC-2*spd* cells after CSC treatment. **a** Lipid ROS level in the GC-2*spd* cells after CSC treatment and after pre-cultured with Fer-1. **b** Iron level in the GC-2*spd* cells after CSC treatment and after pre-cultured with Fer-1. **c** GSH level in the GC-2*spd* cells after CSC treatment and after pre-cultured with Fer-1. **d, e** GPX4 level in the GC-2*spd* cells after CSC treatment and after pre-cultured with Fer-1.*P < 0.05,**P < 0.01 represents the statistical difference compared to the respective group (n = 3)

To investigate the ferroptosis level induced by CSC in the GC-2*spd* cells, GSH, lipid ROS and iron level were measured. GSH level was reduced following treatment with CSC in 24 h, and lipid ROS and iron level were increased (*P* < 0.05). However, the level of them were reduced after co-cultured with Fer-1 (P < 0.05) (Fig. 3a, b, c). Besides, the level of GPX4 protein was reduced after treating with CSC for 24 h, and restored after the cells were treated with CSC and Fer-1(P < 0.05) (Fig. 3d, e). According to these results, CSC-induced toxicity in the GC-2*spd* cells was also contributed by ferroptosis.

## Discussion

Although cigarette smoking is considered as a major risk factor for several human diseases, such as lung cancer and chronic obstructive pulmonary disease (COPD) [33, 34], the effects of smoking on male infertility are controversial. Some studies have reported that cigarette smoking could significantly decrease basic semen parameters [17–19], whereas other studies had shown that cigarette smoking did not affect semen quality [14, 15]. In the present work, a case–control study was conducted between heavy smokers and non-smokers, the results showed that sperm vitality, and sperm progressive motility between the two groups were significantly different. The sperm quality was lower in the group of heavy smokers.

The molecular mechanisms’ underlying smoking-affect semen quality are not clear yet. Smoking has been demonstrated to increase DNA fragmentation and damage in sperm mainly by inducing apoptosis [35, 36]. Caspase-independent apoptosis resulted from oxidative stress mediates this process. However, this mechanism is not solely responsible for affecting semen quality in heavy smokers.

Ferroptosis, a new regulated cell death identified by Brent R. Stockwell’s lab in 2012, is mediated by an iron dependent accumulation of lipid reactive oxygen species (ROS). Study by Li et al. showed that ferroptosis is a pervasive and dynamic type of cell death induced by Oxygen-glucose deprivation and reoxygenation injury in Sertoli cells [37], Elizabeth G explored the contribution of ferroptosis to the demise of germline cells during periods of acute stress in vivo [28]. Previous studies have verified that the lethal lipid peroxidation reaction is accelerated by intracellular iron in the germ cells, whereas other divalent cationic metals have no effect [38, 39].

Based on these discoveries, we present the first findings of ferroptosis in the seminal plasma from heavy smokers. In this study, we found that GSH level was reduced in the group of heavy smokers. However, the level of lipid ROS and iron were increased. These results indicate that the ferroptosis level was increased in the seminal plasma of heavy smokers. For the further proof that the effect on semen quality of heavy smokers by ferroptosis, GC-2*spd* cell line was adopted in this study. After the cell was treated with CSC, the cellular lipid ROS and iron level increased and level of GSH dropped. Fer-1 acts as a lipid ROS scavenger and suppresses ferroptosis specifically [22, 40], and it was usually used as a protector of resisting ferroptosis. According to the results, Fer-1 significantly depressed the cell death and lipid ROS generation caused by CSC in GC-2*spd* cells, this indicated that lipid peroxidation might be involved in CSC-induced cell death. Besides, the level of GPX4 protein, which is the protector of ferroptosis, was also found to be downregulated in GC-2*spd* cell after the cells were treated with CSC. However, level of GPX4 could be restored by treating the cells with CSC and Fer-1 simutaneously. Based on these findings, CSC-induced toxicity in the GC-2*spd* cells was also contributed by ferroptosis. This indicates that smoking is associated with abnormalities of human semen because the ferroptosis induced by lipid peroxidation. The major factors regulating ferroptosis are lipid peroxidation and iron metabolism signaling [21, 22]. Two mechanisms maybe responsible for inducing ferroptosis: (1) the indirect way of inhibiting GSH via the deprivation of the precursor Cys (cystine), as a result of the subsequent influence on the system xc^−^, and (2) inactivating GPX4 directly by compounds such as RSL3 [21, 23]. A variety of inhibitors and inducers of ferroptosis have been reported to regulate the accumulation of lethal lipid reactive oxygen species (ROS) derived from iron metabolism turbulence [22, 41]. In this study, the level of GPX4 protein and GSH level were reduced and the level of MDA and iron were increased. Reduced GSH serves a role in antioxidant defense, and the GSH-GPX4 interaction is critical for the regulation of redox [21]. Depletion of GSH, a basal cofactor of GPX, impairs GPX4 function which can lead to cellular redox state changes and ferroptosis [21]. But the mechanism of ferroptosis induction and the associated signaling pathway responsible for regulating cell death still needs to be further proven.

In conclusion, this study indicates that cigarette smoking is associated with high level of ferroptosis in seminal plasma and affects sperm quality. Accordingly, this finding may be used as a clinical basis for the development of new efficacious treatments for smoking-induced cell death and male infertility.

## Data Availability

Data were obtained from the referenced publications. Further information is available by contacting Dr. Ou at zhanhui-ou@hotmail.com.

## Abbreviations

GSH: Glutathione
ROS: Lipid reactive oxygen species
GPX4: GSH-dependent peroxidase 4
CSC: Cigarette smoke condensate
ATCC: American Tissue Culture Collection
DMEM: Dulbecco modified Eagles media
CCK-8: Cell Counting Kit-8
MDA: Malondialdehyde
Fer-1: Ferrostatin-1
COPD: Chronic obstructive pulmonary disease
